# Identification and Genome Characterization of a Dahlia Common Mosaic Virus Isolate from China

**DOI:** 10.3390/genes14101833

**Published:** 2023-09-22

**Authors:** Jing Wang, Jiaying Zhou, Ming Chen, Zhengnan Li, Shuang Song

**Affiliations:** 1 College of Plant Protection, Northeast Agricultural University, Harbin 150030, China; 2 College of Agriculture, Northeast Agricultural University, Harbin 150030, China; 3 College of Horticulture and Plant Protection, Inner Mongolia Agricultural University, Hohhot 010018, China

**Keywords:** dahlia, high-throughput sequencing, genome, phylogenetic analysis

## Abstract

Dahlia (*Dahlia variabilis*) is a widely cultivated ornamental and medicinal plant in China. Recently, dahlia plants with symptoms of leaf mottling and distortion were collected in Hohhot, Inner Mongolia, China. The presence of dahlia common mosaic virus (DCMV), an unassigned species in the genus *Caulimovirus*, was confirmed by high-throughput sequencing. Three fragments of DCMV Inner Mongolia isolate (DCMV-IN) were PCR-amplified with specific primers, sequenced and assembled into the complete genome sequence with a GenBank accession number of OR494328. The double-stranded circular DNA genome of DCMV-IN consists of 7949 bp and contains six open reading frames (ORFs). Sequence analysis showed that DCMV-IN shared high sequence identities with other DCMV isolates available in the GenBank database. Phylogenetic analysis of DCMV isolates and other representative caulimoviruses based on genome sequence clustered four DCMV isolates to a single branch which was closest to dahlia mosaic virus (DMV). No recombination event was detected among the four DCMV isolates.

## 1. Introduction

Dahlia (*Dahlia variabilis*, genus *Dahlia*, family Asteraceae), also known as Dongyangju and daliju in Chinese, is a perennial herbaceous plant. It possesses not only ornamental but also medicinal value [1]. It has been reported to contain high levels of inulin, which can be used to reduce blood glucose levels for diabetic patients [2]. Dahlia is native to Mexico and is widely cultivated in Yunnan, Guizhou, Inner Mongolia, and other regions of China. Due to the fact that dahlia is usually propagated asexually by vegetative tuberous roots in cultivation, viral infections become a significant threat to dahlias [3]. Dahlia mosaic disease is a widespread and detrimental disease in dahlia with a variety of symptoms such as distortion, mosaic and chlorosis of leaves and overall stunting of plant [4,5]. Three caulimoviruses including dahlia mosaic virus (DMV), dahlia common mosaic virus (DCMV) and DMV-D10 have been reported to be associated with dahlia mosaic disease [6,7,8].

DCMV is an unassigned species of the genus *Caulimovirus* in the family *Caulimoviridae*, which was first reported in the Netherlands in 2008 [9]. It has a wide range of hosts including the members of the family Asteraceae, Brassicaceae, Amaranthaceae and Chenopodiaceae, and it can be transmitted by mechanical inoculation and aphids in a non-persistent manner [8]. DCMV has an approximately 8 kb circular double-stranded genomic DNA [10]. The genome organization of DCMV is consistent with that of typical members of the genus *Caulimovirus*, consisting of at least six open reading frames (ORFs) (ORF I–VI) that successively encode movement protein (MP), aphid transmission factor (ATF), putative DNA-binding protein (DNAb), capsid protein (CP), polymerase polyprotein (PP)-containing protease, reverse transcriptase and RNAse H and an inclusion body protein (IB) [9,11].

**Figure 1:**
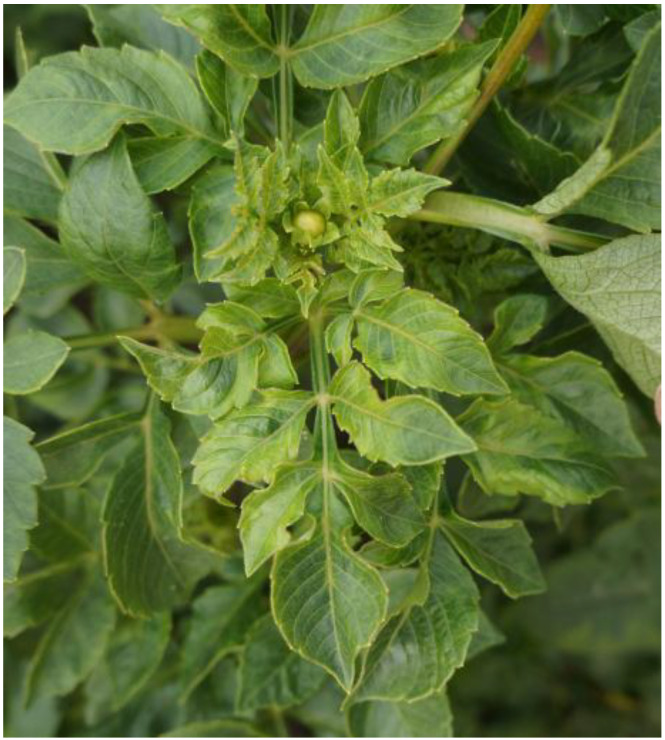
The observed dahlia plants with symptoms of leaf mottling and distortion.

Recently, dahlia plants displaying virus-like symptoms of leaf mottling and distortion (Figure 1) were discovered in the campus of Inner Mongolia Agricultural University. The presence of DCMV infection in dahlia plants was confirmed through high-throughput sequencing (HTS) and PCR confirmation. Furthermore, the complete genome sequence of the virus was sequenced and analyzed.

## 2. Materials and Methods

### 2.1. Sampling of the Virus Source

In July 2022, Dahlia samples showing obvious virus-like disease symptoms of leaf mottling and distortion (Figure 1) were observed in the campus of Inner Mongolia Agricultural University in Hohhot, Inner Mongolia, China. Leaf samples were collected, immediately frozen using liquid nitrogen and transferred to −80 °C for further research.

### 2.2. High-Throughput Sequencing (HTS) and Data Analysis

To identify the virus infecting dahlia, total RNA was extracted from the pooled symptomatic leaf samples from three different plants, with each sample weighing approximately 200 mg. TRIzol reagent (Invitrogen, Carlsbad, CA, USA) was used for the extraction following the manufacturer’s instructions. Ribosomal RNA (rRNA) was removed from the total RNA using a Ribo-Zero rRNA Removal Kit (Epicentre, Madison, WI, USA), and the rRNA-depleted RNA was used to construct a cDNA library using a TruSeq RNA Sample Prep Kit (Illumina, San Diego, CA, USA). The prepared library was sequenced using an Illumina NovaSeq 6000 platform with a read length of 2 × 150 bp. The raw data obtained from the original image were processed by trimming adapter sequences and getting rid of low-quality reads of length < 75 bp using the software Trimmomatic v.0.36 [12]. The taxonomy of the clean reads was determined by the software Kraken2 v.2.0.6 [13]. The obtained virus-associated clean reads were then assembled de novo into contigs using MegaHit with ‘—min-contig-len 500′ parameters [14]. The obtained contigs were subjected to BLAST analysis against the NCBI nucleotide (nt) sequences database and NCBI non-redundant protein (nr) database, with an e-value cutoff of 10^−5^ [15].

### 2.3. Determination of DCMV Genome

To verify the presence of DCMV in the sample identified by HTS and determine its complete genome sequence, total DNA was extracted from the pooled symptomatic leaf samples used for HTS, using EasyPure Plant Genomic DNA Kit (TransGen, Beijing, China). PCR was performed using three specific primer pairs designed based on the DCMV contigs obtained from HTS (Table 1). The PCR reaction was carried out in a total volume of 20 μL containing 1.0 μL of total DNA; 10.0 μL of KOD One^TM^ PCR Master Mix (Toyobo, Shanghai, China); and 0.6 μL of 10 μM corresponding to upstream and downstream primers, with the following conditions: 35 cycles of denaturation at 98 °C for 10 s, annealing at 55 °C for 5 s and elongation at 68 °C for 200 bp/s. The PCR products were examined by 1% agarose gel electrophoresis, purified using EasyPure Quick Gel Extraction Kit (TransGen, Beijing, China), cloned into the pMD18-T simple vector (TaKaRa, Dalian, China) and sequenced by the Sanger method. For each amplicon, three independent clones were sequenced. The complete genome sequence was assembled using Vector NTI software v.10.1.1 (Invitrogen, Carlsbad, CA, USA) based on overlapping fragments, and the ORFs were predicted using ORFfinder (https://www.ncbi.nlm.nih.gov/orffinder (accessed on 1 June 2023)). The online tool MotifFinder (https://www.genome.jp/tools/motif/ (accessed on 1 June 2023)) was used to identify conserved motifs of the encoded proteins.

### 2.4. Genetic Variation, Phylogenetic and Recombination Analysis

The assembled genome sequence was subjected to BLAST analysis in GenBank database (https://blast.ncbi.nlm.nih.gov/Blast.cgi; accessed on 20 June 2023). All DCMV genome sequences available in GenBank and representative *Caulimovirus* species were retrieved and analyzed together with those of the DCMV isolate determined in this work. Pairwise nucleotide and amino acid sequence identities were determined using SDT 1.0 [16]. All of the circular genomes were linearized starting at the coding region of MP [11]. Multiple sequence alignments were performed using the ClustalW algorithm in MEGA11 [17]. Using MEGA11, maximum-likelihood (ML) phylogenetic trees were constructed based on the complete genome sequences with 1000 bootstrap replicates. Genetic distances were calculated by the General Time Reversible (GTR) model which was selected as the best-fit substitution model through the ‘Find Best DNA/Protein Models’ program, and a discrete γ distribution was used to model evolutionary rate differences among sites [17]. Seven of the recombination detection methods, including RDP, Geneconv, Chimera, BootScan, MaxChi, SiScan and 3Seq, implemented in RDP4 software package v.3.44 [18], were used to identify potential recombination events. Only the events supported by at least five of the seven methods with *p* < 0.01 were accepted.

## 3. Results and Discussion

### 3.1. Virus Identification by HTS

A cDNA library was constructed from pooled leaf tissues of three symptomatic dahlia plants and sequenced using Illumina NovaSeq 6000 platform. A total of 36,031,741 clean reads with Q20 of 96.91% and Q30 of 91.59% were obtained after trimming adapter sequences and discarding low-quality reads. The virus-associated clean reads were assembled de novo into 2213 contigs ranging from 141 to 6630 bp (Supplementary Table S1). Further BLAST analysis showed that, among these contigs, the longest one with a length of 6630 bp covered 83.4% (6630/7949) of the genome of DCMV New Zealand isolate DCMV-NZ (Accession No. JN032736) [11] and shared a 99.46% pairwise identity. Therefore, the DCMV isolate associated with dahlia mosaic disease that was collected in Inner Mongolia of China in this study was named DCMV-IN.

### 3.2. Determination and Characterization of the DCMV-IN Genome

Three fragments with lengths of 3162 bp, 2856 bp and 2343 bp were PCR-amplified from total DNA with specific primers DCMV-F1/DCMV-R1, DCMV-F2/DCMV-R2 and DCMV-F3/DCMV-R3, respectively. The complete genome sequence of DCMV-IN was assembled based on overlapping regions and deposited in the NCBI GenBank database under accession number OR494328. DCMV-IN genome consisted of 7949 bp containing six ORFs. ORF I (1–969 nt) encoded MP of 322 aa. The transport domain GNLCYGKFMFTVY associated with cell-to-cell movement for caulimoviruses was found from 166 to 178 aa, which was consistent with other DCMV isolates although the cysteine was replaced by alanine for other caulimoviruses [9]. ORF II (962–1453 nt) encoded ATF of 163 aa, and the essential motif for aphid transmission through interaction between ATF and virus particle (IXG) was observed at the C-termianl [19]. DNAb of 120 aa and CP of 505 aa were encoded by ORF III (1450–1812 nt) and ORF IV (1797–3314 nt), respectively. An RNA-binding domain (CX2CX4HX4C) CWICAEEGHY ANEC was identified at 441–454 aa of CP [20]. ORF V (3311–5332 nt) encoded a polyprotein of 673 aa. Three conserved motifs in caulimovirus replicases including aspartyl protease, reverse transcriptase and ribonuclease H at aa positions 76–132, 290–447 and 511–645, respectively, were identified by MotifFinder in the polyprotein. ORF VI (5459–6973 nt) encoded IB of 504 aa, and a conserved motif of caulimovirus viroplasm was identified at 144–181 aa.

### 3.3. Comparison of DCMV Genome Sequences

BLAST analysis retrieved the complete genome sequences of three DCMV isolates, including one isolate from New Zealand (DCMV-NZ, JN032736) [16], one from Japan (DCMV-JP, LC625373) [21] and one from Beijing, China (DCMV-BJ, KX098538), and one near-full-length genome which covered all ORFs from Taiwan, China (DCMV-TW, AB740270-AB740275) [22]. DCMV-IN shared high genome sequence identities of 99.4%, 99.3% and 99.4% with DCMV-NZ, DCMV-JP and DCMV-BJ, respectively (Table 2). The pairwise nucleotide sequence identities between DCMV-IN and other DCMV isolates ranged from 97.9 to 99.1%, 97.2 to 99.6%, 98.4 to 99.7%, 98.8 to 99.7%, 99.1 to 99.5% and 99.3 to 99.6% for ORF I–VI, respectively. The amino acid sequence identities were 97.5–98.8%, 96.3–99.4%, 95.8–99.2%, 98.2–99.8%, 99.1–99.7% and 98.8–99.8% for ORF I–VI, respectively.

### 3.4. Phylogenetic and Recombination Analyses

**Figure 2:**
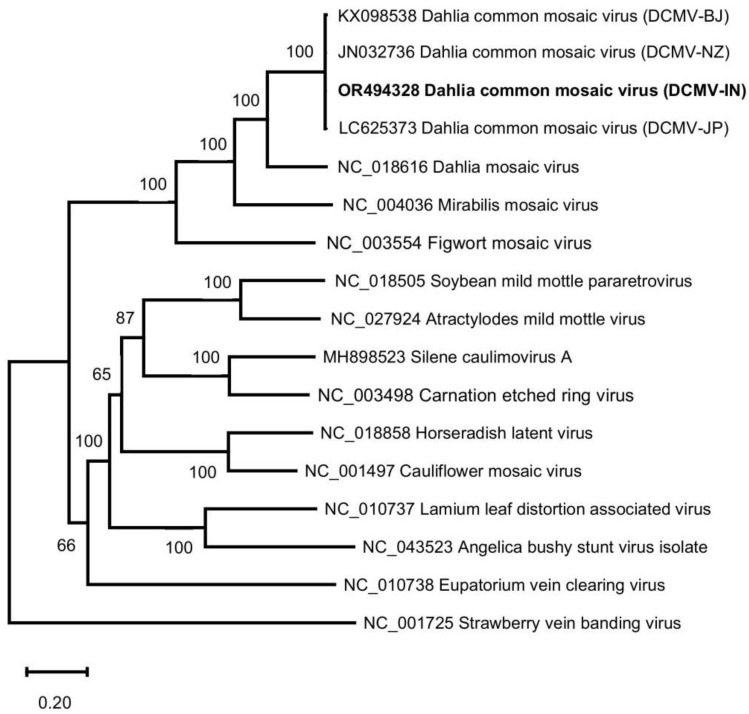
Phylogenetic analysis by maximum-likelihood (ML) method based on complete genome sequences of DCMV and other caulimoviruses. DCMV-IN determined in this study is marked in bold.

The complete genome sequences of four DCMV isolates containing DCMV-IN in this work, and other 13 representative caulimoviruses, were subjected to phylogenetic analysis. In the constructed phylogenetic ML tree (Figure 2), the four DCMV isolates (DCMV-IN, DCMV-BJ, DCMV-NZ and DCMV-JP) were clearly clustered in a single branch. The cluster of DCMV formed a clade together with DMV, mirabilis mosaic virus (MMV) and figwort mosaic virus (FMV), and DCMV was closest to DMV. The phylogenetic tree based on complete genomes in this work showed a similar topology with those based on ORF II, ORF III, ORF IV and ORF VI [4,9]. No putative recombination event was identified from the genome of the four DCMV isolates.

Both the results of pairwise alignment and phylogenetic analysis showed that the reported DCMV isolates around the world had low genetic diversity, suggesting that asexual propagation of dahlia plants by vegetative tuberous roots may be the main route of DCMV transmission. This work not only provides a background for an in-depth study on the epidemic and evolution of DCMV, but also highlights the necessity of virus detection in propagating materials for prevention and control of dahlia mosaic disease.

## Figures and Tables

**Table 1:** Primers used for determination of DCMV genome.

Primer	Sequence (5′-3′)	Position ^a^
DCMV-F1	CAGTCTGGAATCGATACACC	409–428
DCMV-R1	TCTTGGTTAGCCACTGTAACCTG	3548–3570
DCMV-F2	AGGAAAGTTATCCTTTAAGGGA	3418–3439
DCMV-R2	GGACCGATTATGAGAAAGCTTC	6252–6273
DCMV-F3	ACCAGAAAACTCTCAACAGGA	6118–6138
DCMV-R3	ATAGCACAAGTTGCCTTTTGCTG	488–510

^a^ Binding position relative to the genome sequences of DCMV-IN (GenBank No. OR494328).

**Table 2:** Pairwise nucleotide (upper) and amino acid (lower) sequence identities of DCMV-IN with four other DCMV isolates available in GenBank.

Virus Isolate	Genome	ORF I	ORF II	ORF III	ORF IV	ORF V	ORF VI
DCMV-NZ	99.4 -	99.1 98.8	99.6 98.8	98.4 95.8	99.7 99.8	99.4 99.6	99.4 99.4
DCMV-JP	99.3 -	98.8 98.8	99.6 99.4	98.6 96.7	99.4 99.0	99.4 99.7	99.6 99.8
DCMV-BJ	99.4 -	99.1 98.8	99.6 99.4	99.2 95.8	99.3 99.4	99.5 99.6	99.5 99.4
DCMV-TW	NA -	97.9 97.5	97.2 96.3	99.7 99.2	98.8 98.2	99.1 99.1	99.3 98.8

## Data Availability

The sequence data have been submitted to GenBank databases under accession number OR494328. Addresses are as follows: GenBank http://www.ncbi.nlm.nih.gov (accessed on 1 May 2023).

## Supplementary Materials

The following supporting information can be downloaded at: https://www.mdpi.com/article/10.3390/genes14101833/s1, Table S1: Summary of the contigs de novo assembled from virus-associated clean reads.
